# Strengthening the patient perspective in quality development: a mixed-methods observational study on suitability of the PROMIS GH-10 in Swiss inpatient rehabilitation

**DOI:** 10.1186/s12913-026-15291-7

**Published:** 2026-08-12

**Authors:** Marie Utsch, Gavin Brupbacher, Gaia Garuffi, Anke Scheel-Sailer, Stephan Tobler, Manuela Marquardt

**Affiliations:** 1 Institut für Medizinische Soziologie und Rehabilitationswissenschaft, Charité – Universitätsmedizin Berlin, Berlin, Germany; 2 OBERWAID AG, St. Gallen, Switzerland; 3 Department of Consultation-Liaison Psychiatry and Psychosomatic Medicine, University Hospital Zurich, Zurich, Switzerland; 4 ANQ, Bern, Switzerland; 5 Centre for Rehabilitation and Sport Medicine, Inselgroup, University Bern, Bern, Switzerland

**Keywords:** Patient-reported outcome measures, Quality development, PROMIS GH-10, Generic health-related quality of life, Switzerland, Inpatient rehabilitation, Feasibility, Mixed-methods

## Abstract

**Objective:**

Healthcare systems increasingly use patient-reported outcome measures (PROMs) to complement traditional clinical and performance-based outcomes by capturing health outcomes from the patient perspective and supporting quality improvement and patient-centred care. In Switzerland, the National competence centre for quality measurements in hospitals and clinics ANQ performs the external quality development program. We conducted a nationwide pilot study in inpatient rehabilitation using the generic PROMIS GH-10 to assess its suitability as an outcome for quality development by evaluating its responsiveness and feasibility of implementation in everyday clinical practice.

**Methods:**

Our study applied a mixed-method observational design. In a multi-stage expert-based selection process, the PROMIS GH-10 was selected for piloting. In total, 28 rehabilitation clinics recruited patients during the study period and asked them to fill in the PROMIS GH-10 digitally or paper-based at admission and discharge. After the survey, healthcare professionals (HCPs) were asked to document the level of assistance required by patients. Mean T-scores and effect sizes (Cohen’s d) were calculated. Assistance requirements were analysed using descriptive statistics. Additionally, qualitative interviews were completed with HCPs to assess facilitating factors and barriers to implementation.

**Results:**

A sample of 2,083 patients was included, resulting in 1,217 complete pre-post measurements. Mean T-scores (SD) for PROMIS GH-10 subscales physical health (PHS) and mental health (MHS) significantly improved (*p* < 0.001) from admission to discharge (total sample PHS: 39.3(7.4); 44.4(7.6); d = 0.68; MHS: 44.9(8.0); 48.7(7.5); d = 0.48). Effect sizes on PHS ranged from d = 0.50–0.95 and on MHS from d = 0.33–1.19. Overall, 41% of patients required assistance in answering the PROM. Barriers to implementation from HCP perspective included uncertainties about the benefit of a generic PROM and the lack of staff resources. Facilitators were the questionnaire’s brevity and the value of including the patient perspective.

**Conclusions:**

PROMIS GH-10 showed significant pre-post effects on physical and mental health in Swiss inpatient rehabilitation. Nonetheless, barriers such as increased time needed for assistance in special patient groups should be considered before national implementation.

**Supplementary information:**

The online version contains supplementary material available at 10.1186/s12913-026-15291-7.

## Introduction

Healthcare systems worldwide are obliged to assess their quality to initiate improvement strategies with the goal of increasing quality and saving costs. This includes the evaluation of diagnostic and treatment processes, structural and health care professional (HCP) resources, and the patient perspective. The use of patient-reported outcome measures (PROMs) is a promising approach to include the patient’s perspective in such assessments. PROMs make it possible to record patients’ own perceptions of their health-related outcomes in a standardised manner.

PROMs are becoming an increasingly important measure of treatment quality enabling focused quality development [1–5]. Recently, PROMs have even been used in cross-country comparisons for quality development [6].

In Switzerland, the Federal Office of Public Health has identified patient-centred care as a key area of action within its current quality strategy [7]. Reflecting this emphasis, the Rehabilitation Quality Committee (QA Rehabilitation) of the Swiss national competence centre for quality measurements in hospitals and clinics. ANQ has been refining the rehabilitation measurement plan since 2019, prioritising the patient perspective based on the rehabilitation quality development model. This approach broadens the focus beyond solely functional and performance-based outcomes by incorporating patient-reported outcomes, which systematically capture the patients’ own perspectives on their health and recovery.

Among the typical constructs tested with PROMs are symptoms, functional status, and health-related quality of life (HRQOL). There are now numerous generic as well as disease-specific measurement instruments for recording HRQOL with a wide range of overlapping content and different focal points depending on the development context. Common to all HRQOL measures is their multidimensional construct, considering physical, mental, and social subjective health aspects that are important to patients’ everyday lives.

In addition to clinical parameters, HRQOL is becoming increasingly relevant as an outcome parameter in medical care research [8] particularly in the case of chronic diseases where complete recovery cannot be achieved [9]. Since rehabilitation follows the framework of the International Classification of Functioning, Disability and Health (ICF), which emphasises functioning, participation, and contextual factors [10], HRQOL is particularly well-suited as an outcome measure in this setting. Improving HRQOL is therefore a central treatment goal of rehabilitation, in addition to improving functioning and participation.

PROM implementation is still difficult or missing in rehabilitation for several reasons, for instance issues with data collection or patient group heterogeneity [11]. A report on PROMs in international comparison identified further challenges such as difficulties in completing the questionnaires or the high administrative burden and lack of benefits in care [12], all of which relate to the overall feasibility of the instrument in routine practice.

Therefore, in external quality assurance and development, most outcomes are still routinely measured using administrative data or clinician-rated instruments.

The international literature on generic PROMs in rehabilitation is sparse. International implementation studies highlight that, while harmonisation is desirable for comparability, national contexts differ significantly and require tailored solutions [13]. Consequently, piloting is recommended to gather insights and experiences prior to full-scale implementation [14].

The primary goal of this pilot project was to strengthen the patient perspective in Swiss inpatient rehabilitation on the national level. We analysed this aim through two complementary aspects: first, we assessed the suitability of the PROMIS GH-10 quantitatively by examining its responsiveness; second, we evaluated the feasibility, focussing on practical implementation aspects in everyday clinical practice to inform a potential implementation process.

## Methods

We conducted a mixed-methods observational study on the national level in inpatient rehabilitation across all nine rehabilitation domains in Switzerland. As defined in the national DefReha paper, the ANQ distinguishes nine rehabilitation domains: geriatric, internal medicine, cardiac, musculoskeletal, neurological, oncological, paraplegic, psychosomatic, and pulmonological rehabilitation [15]. These domains are based on medically defined service types in the national health system to support national planning and tariffing, rather than being strictly diagnosis-based. Due to the distinct medical characteristics and patient populations within each rehabilitation domain, all analyses were conducted according to rehabilitation domains, consistent with routine quality assurance practices.

To address the responsiveness of the PROMIS GH-10 as a HRQOL measure, data were collected using quantitative methods. Feasibility was examined through a parallel collection of both quantitative and qualitative data.

Reporting of our study followed the Strengthening the Reporting of Observational Studies in Epidemiology checklist [16], the Good Epidemiological Practice guidelines [17], and the MMR-RHS Checklist [18].

### Setting

This pilot project was conducted as a voluntary additional measurement in the setting of the ANQ’s regular national quality assessment in inpatient rehabilitation, whose results are published yearly on the ANQ’s website (http://www.anq.ch).

In a multi-stage selection process, the QA Rehabilitation decided to pilot a suitable PROM. The expert-consented criteria to define a subset of suitable measures were: generic, electronically usable, validated in all three Swiss languages (German, French and Italian), psychometric properties, length, and costs. We carried out research into common HRQOL instruments and their use in international quality assurance systems. In addition to the expert-consented selection criteria, the QA Rehabilitation considered the PROMIS GH-10 as the most appropriate PROM due to its dual physical and mental scoring, and broad international use, including several ICHOM standard sets [19].

To realise the PROMIS GH-10 Data Project Network, the following project partners were brought together, led by the ANQ. The Bern University of Applied Sciences, MIDATA, and Brightfish were responsible for the measurement logistics of the electronic data collection. The semi-structured interviews were realised with a project partner, socialdesign. The Charité - Universitätsmedizin Berlin was responsible for data monitoring, data analysis and reporting.

A total of 28 inpatient rehabilitation clinics with 67 rehabilitation domains from the three language regions of Switzerland (German, French, Italian) took part in this study.

All pilot clinics were trained in the planned processes of the pilot project (e.g. implementation and use of PROMIS GH-10, use of tablets to complete the questionnaire) and were supported throughout the whole data collection process, which was accompanied by data monitoring. However, processes were flexible enough to allow for individual approaches in the clinics.

Data was collected between April and December 2023. Initially, the clinics obtained informed consent from patients. The clinics were instructed to carry out the admission measurement (T1) within the first three days after admission to inpatient rehabilitation and the discharge measurement (T2) within the last three days of inpatient rehabilitation (admission/discharge day included, analogous to the ANQ’s rehabilitation measurement plan). The typical rehabilitation period is 21 days. However, in some rehabilitation domains, such as paraplegia, the average length of rehabilitation stays is much longer.

The questionnaire was completed either on a digital device or on paper. By giving their consent to participate, patients also agreed to the matching of their data with the regular ANQ measurement data, including sociodemographic and administrative data (submitted to the Federal Statistical Office) and the Cumulative Illness Rating Scale (CIRS) [20], a scale for morbidity assessment.

### Sample size

We conducted a power calculation using G-Power to determine the required number of cases per rehabilitation domain. A one-sided t-test for paired samples was applied, with an alpha level of 0.05 and a beta level of 0.95, assuming a medium effect size for most rehabilitation domains on both subscales (PHS and MHS) of the PROMIS GH-10. The calculations indicated that, for medium effect sizes (d: 0.4 to 0.6), between 32 and 70 complete pre-post measurements with the PROMIS GH-10 are necessary per rehabilitation domain.

In consultation with the QA Rehabilitation, the minimum requirements for the pilot sample were established. These included the participation of 5 clinics, with adequate representation from each language region (preferably 2 clinics from the German-speaking region, 2 from the French-speaking region, and 1 from the Italian-speaking region of Switzerland). Ideally, each clinic should provide at least 30 complete cases, with both pre- and post-intervention data.

### Participants/eligibility criteria

The pilot clinics actively recruited patients between the beginning of April and the beginning of December 2023. Eligibility criteria for inclusion in the dataset followed the standards defined in the national measurement plan of the ANQ for inpatient rehabilitation cases and included:

- Age 18 years or older
- Completion of inpatient rehabilitation treatment (>7 days)

### Measurements

The PROMIS GH-10 consists of ten generic formulated items covering physical, mental, and social aspects of subjective health [9] (see Supplementary material 1). Each item is scored on a 5-point scale, except Global07, which is scored on an 11-point numerical scale and recoded to a 5-point scale by the recommendations outlined in the PROMIS-GH Scoring Manual [21]. For three questions, an observation period of the previous seven days is addressed, while the remaining seven questions relate to the current condition at the time of the survey e.g. “In general, how would you rate your physical health?” or “How would you rate your fatigue on average?”

After the PROMIS GH-10 assessment, HCP additionally recorded whether the patient required assistance with filling out the survey. Possible assistance requirements were: reading out questions/answers, data entry on behalf of the patient, answering questions about the questionnaire, and other (with a free text field). In addition, the free text field answers were grouped and a new category ‘multiple support’ emerged. In the case of patients who refused the test despite giving consent, the HCP documented the reasons for this. Possible answers were no interest, physical/motor impairment, cognitive impairment, patient not encountered, survey not available in preferred language, other (with free text field). Drop-outs, in the sense of unexpected discontinuation of treatment, were instructed to document by the HCP in the open field.

Information on the rehabilitation domain, mode of administration of the survey (electronic or paper-based), language version and dates were available for both measurements.

As part of the data monitoring, field notes were made on problems and questions that arose in the clinics.

### Statistical analysis

Availability and completeness of measurements at admission or discharge were analysed descriptively. The pre-post effects were analysed for complete cases only. Two summary scores were calculated, based on eight of the 10 PROMIS GH-10 items: the physical health summary score (PHS) and the mental health summary score (MHS). The remaining two items were not necessary for the calculation of the score. To calculate the scores using the T-score table, the point values [1–5] were added to a raw total score [5–9, 11–14, 16, 17, 21–23] and then assigned to the corresponding *T*-value. The following applied to the interpretation of the values: A *T*-value of 50 corresponds to the average of the general population. *T*-values of 40 and 60 are one standard deviation below and above the population average, respectively. A higher score indicates better global mental/physical health [5, 19].

In addition to the T-score mean values for admission and discharge and other distribution parameters, t-tests and effect sizes (Cohen’s d) were calculated using pooled standard deviation [19]. Effect sizes were calculated for each rehabilitation domain and are reported descriptively; no formal statistical comparisons between domains were planned.

The effect size was interpreted as a standardised mean difference: around 0.2 was considered a small effect, around 0.5 a medium effect, and 0.8 or above a strong effect [22].

The data on assistance requirements was analysed using descriptive statistics. For this purpose, all cases, complete and incomplete, were analysed.

### Qualitative methods and analysis

To assess the PROMIS GH-10’s feasibility in clinical practice, we conducted semi-structured individual interviews and group interviews with HCPs, who were involved in carrying out the study, regardless of their profession.

The experienced Swiss survey institute socialdesign organised and carried out the qualitative interviews. The qualitative guide was conducted in accordance with a set of 11 guiding questions, which were complemented by a series of more detailed inquiries pertaining to the following subject areas (see Supplementary material 2):

- Challenges and potential solutions
- Effort and benefits
- Practicability and acceptance
- Concept of PROMs
- Implementation

Participants were recruited via the pilot clinics. Depending on capacity and preference, individual interviews (interview with one participant) or group interviews (interview with two or more participants) were offered. Apart from a round of introductions in the group interviews, the procedure was the same. All qualitative interviews were done online and audio-recorded. All recordings were pseudonymised, transcribed and translated into German by socialdesign and sent to Charité.

We analysed the materials of all interviews using qualitative content analysis according to Kuckartz with MAXQDA [23]. A deductive and inductive approach was used to create categories. For the analysis, deductive main categories were first formed based on the research question and the interview guide, which were then inductively supplemented with material-based subcategories. This structured and iterative coding process, which involved constant comparison across roles and rehabilitation fields, allowed us to identify facilitating factors, barriers, and implementation suggestions of the PROMIS GH-10 in clinical practice.

## Results

**Fig. 1:**
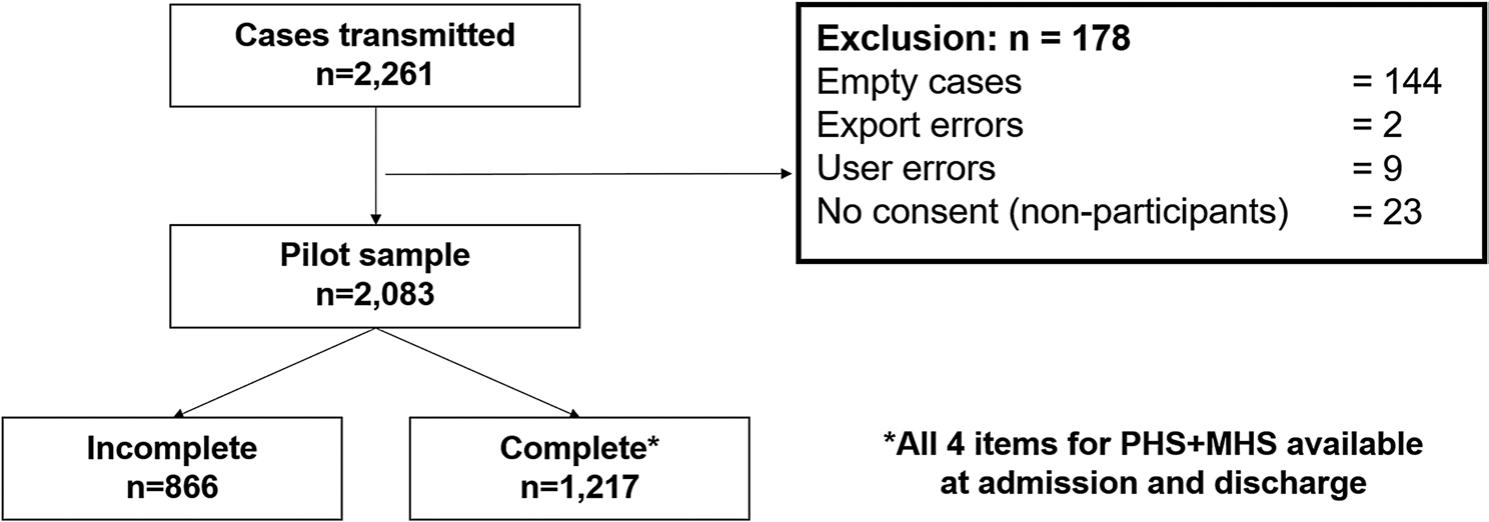
Flow chart

A total of 2,261 cases were transmitted from the pilot clinics, of which 178 were excluded (Fig. 1). This resulted in a final database of 2,083 cases, divided into 866 incomplete and 1,217 complete cases. The sample description of the matched cases can be found in Supplementary material 3. The pilot sample was largely comparable to the regular ANQ measurement sample in terms of the distribution of characteristics, except for the length of stay in paraplegic rehabilitation.

**Fig. 2:**
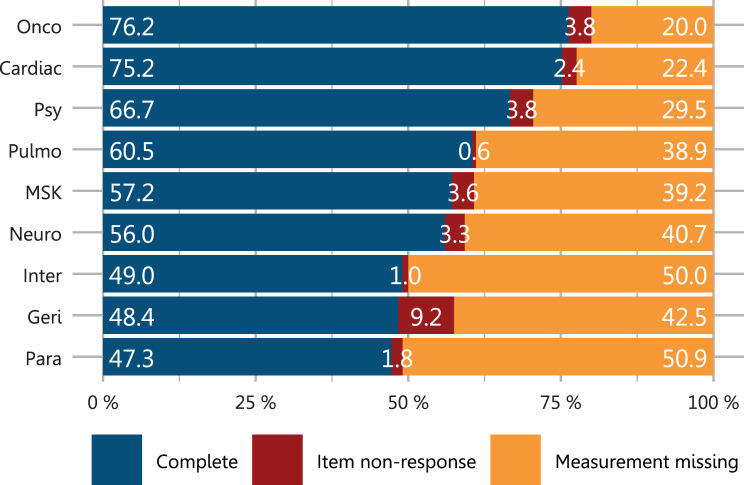
Data quality by rehabilitation domain

The proportion of complete cases for all rehabilitation domains was 58.4%. The proportion of complete cases was comparatively higher in the oncological (76.3%), cardiological (75.2%), and psychosomatic (66.7%) rehabilitation domains, whereas the internal medicine (49.0%), geriatric and paraplegiological (47.3%) rehabilitation domains had a lower rate (Fig. 2).

The majority of all incomplete cases (*n* = 866, 41.6%) had completely missing measurements at either admission or discharge (*n* = 787, 37.8%). Only 3.8% (*n* = 79) were incomplete due to item-non-response, although this proportion was higher in geriatric rehabilitation (9.2%). A detailed analysis of the incomplete cases showed that most of the measurements were complete at admission (*n* = 735, 84.9%) and that most cases were incomplete at the discharge measurement.

For some of the missing measures, HCPs documented reasons (*n* = 168, 21.3%). In 41.1% of these cases, the patient could not be encountered, followed by other reasons (27.4%) such as the patient being transferred and a lack of interest (18.5%). Cognitive (9.5%) and physical (2.4%) impairments accounted for a small proportion of the reasons for not taking the test. In only two cases (1.2%) the reason for non-response was that the survey was not available in the preferred language. In the open-ended information on cases with missing measurements, 40 cases were reported to have discontinued the rehabilitation treatment (drop-outs).

**Fig. 3:**
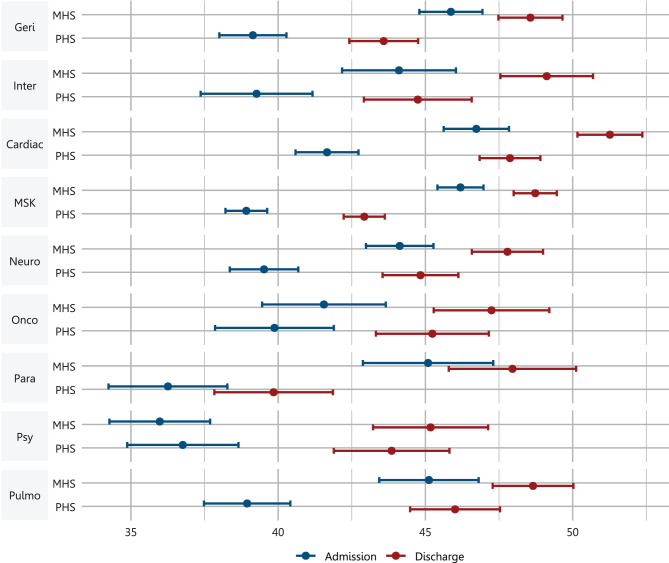
Pre-post scores according to rehabilitation domain

Cases with complete admission and discharge measurements for both PROMIS scores (PHS, MHS) were used for the analysis (*n* = 1,217). Figure 3 shows the mean T-score and associated 95% confidence intervals of the MHS and PHS for the admission and discharge measurements by rehabilitation domain. The corresponding effect sizes can be found in Table 1.

**Table 1:** PROMIS GH-10 T-score mean values and effect sizes for PHS and MHS by rehabilitation domain

rehabilitation domain	score	admission		discharge		n	T	p	Cohen’s d
		mean	SD	mean	SD				
geriatric	PHS	39.1	7.0	43.6	7.2	148	7.6	<0.001	0.63
	MHS	45.9	6.6	48.6	6.7	148	5.6	<0.001	0.41
internal medicine	PHS	39.3	6.8	44.7	6.5	51	5.8	<0.001	0.83
	MHS	44.1	6.9	49.1	5.6	51	6.5	<0.001	0.80
cardiac	PHS	41.7	7.4	47.9	7.2	188	12.2	<0.001	0.85
	MHS	46.7	7.7	51.3	7.6	188	9.8	<0.001	0.59
musculoskeletal	PHS	38.9	7.2	42.9	7.1	395	11.3	<0.001	0.56
	MHS	46.2	7.9	48.7	7.4	395	7.8	<0.001	0.33
neurological	PHS	39.5	7.3	44.8	8.1	154	9.7	<0.001	0.69
	MHS	44.1	7.2	47.8	7.6	154	6.5	<0.001	0.49
oncological	PHS	39.9	7.9	45.2	7.5	61	7.2	<0.001	0.70
	MHS	41.6	8.2	47.2	7.7	61	6.6	<0.001	0.72
paraplegic	PHS	36.3	7.2	39.8	7.2	52	3.6	<0.001	0.50
	MHS	45.1	8.0	48.0	7.8	52	3.2	0.002	0.36
psychosomatic	PHS	36.8	7.9	43.9	8.2	70	9.6	<0.001	0.88
	MHS	36.0	7.2	45.2	8.2	70	11.3	<0.001	1.19
pulmonological	PHS	38.9	7.3	46.0	7.6	98	11.0	<0.001	0.95
	MHS	45.1	8.4	48.7	6.8	98	5.4	<0.001	0.46
total	PHS	39.3	7.4	44.4	7.6	1217	25.7	<0.001	0.68
	MHS	44.9	8.0	48.7	7.5	1217	19.7	<0.001	0.48

PHS physical health summary score / MHS mental health summary score of the PROMIS GH-10; Cohen’s d: 0.20–0.49 small effect; 0.5–0.79 medium effect; ≥0.80 strong effect

The mean PHS at admission was between 38.9 and 39.9 in most rehabilitation domains, which was around one standard deviation below the population average. At discharge, the T-scores of the PHS in most rehabilitation domains varied between 42.9 and 46.0 and were thus much closer to the norm. Descriptively, paraplegiological rehabilitation had a lower mean discharge score (39.8), while cardiac rehabilitation had a higher score (47.9) than the other rehabilitation domains.

The distribution of the MHS also differed descriptively across the various rehabilitation domains. In most rehabilitation domains, the MHS at admission was between 44.1 and 46.7 and thereby somewhat closer to the norm than the PHS. At the time of discharge, the average MHS was between 47.2 and 49.1 and thus almost corresponded to the population average. Cardiac rehabilitation showed a descriptively higher mean T-score (51.3) and in psychosomatic rehabilitation slightly lower (45.2) than in the other rehabilitation domains.

Effect sizes are reported descriptively by domain, without formal statistical comparisons between groups. There were statistically significant pre-post effects across all rehabilitation domains for PHS and MHS (*p* < 0.001). However, in paraplegiological rehabilitation, the pre-post effect in MHS differed slightly, reaching statistical significance at the 99% confidence level (*p* = 0.002). The average effect size for the PHS was 0.68, indicating a medium effect. For the MHS, the average effect size was 0.48, indicating a small effect (see Table 1).

When differentiated by rehabilitation domain, PHS consistently showed medium to high effect sizes. The pre-post effects were strongest in internal, cardiac, psychosomatic and pulmonary rehabilitation. The other rehabilitation domains showed medium effect sizes for the PHS, with paraplegiological and musculoskeletal rehabilitation showing the lowest average effects of all rehabilitation domains.

For the MHS, the pre-post effect was the strongest in psychosomatic rehabilitation, followed by internal medicine rehabilitation. Medium effects were found in oncological and cardiac rehabilitation. Neurological, pulmonary, geriatric, paraplegiological and musculoskeletal rehabilitation showed small pre-post effects on the MHS.

**Fig. 4:**
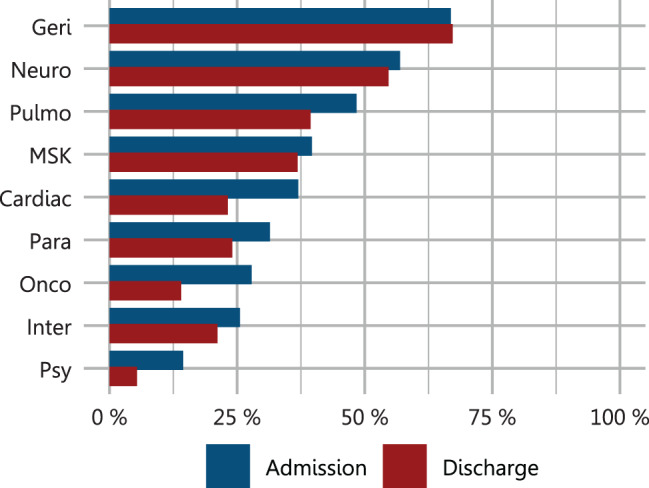
Proportion of cases with assistance requirements by rehabilitation domain for admission and discharge measurement. Assistance requirements include: reading out questions/answers, ticking on behalf of the patient, answering questions about the questionnaire, other

Figure 4 shows the proportion of cases with assistance requirements by rehabilitation domain for admission and discharge measurements (based on all available assessments: admission *n* = 2,015; discharge *n* = 1,290).

Overall, assistance requirements were documented for 41.0% of patients at both time points (*n* = 1,355 of 3,305). Marked differences in assistance requirements were observed across rehabilitation domains: geriatric rehabilitation had the highest rates (admission: 66.9%; discharge: 67.2%), while psychosomatic rehabilitation had the lowest (admission: 14.4%; discharge: 5.4%). Table 2 shows the distribution of the categories of assistance requirements.

**Table 2:** Distribution of the categories of assistance requirements

Category	n	%
reading questions or answers aloud	698	51.5
recording responses on behalf of patients	270	19.9
answering questions about the questionnaire	157	11.6
multiple support	205	15.1
Other	25	1.8

Assistance needs were slightly lower at discharge compared to admission across most rehabilitation domains.

A subgroup analysis examining the requirements for assistance, the completeness of the data, and the mode of administration at both time points did not reveal a clear pattern within the rehabilitation domains. In the overall sample, 38.3% of the complete cases required assistance, compared to 48.3% of the incomplete cases. However, in some rehabilitation domains, the ratio was reversed. Concerning the mode of administration, HCPs documented assistance requirements for 28.1% of the paper-based measurements, compared to 49.4% of the electronic questionnaires.

### Qualitative results

One group interview with three participants, three group interviews with two participants each and four individual interviews were conducted. A total of 13 HCPs from 10 clinics, working in different functions (quality management (four), health care professionals: medical doctors (three), nursing (one) and therapy (one), administration (three), research (one)) and in different rehabilitation fields, were interviewed. Of these, four were French-speaking and nine were German-speaking. The interviews lasted between 30 and 90 minutes.

**Fig. 5:**
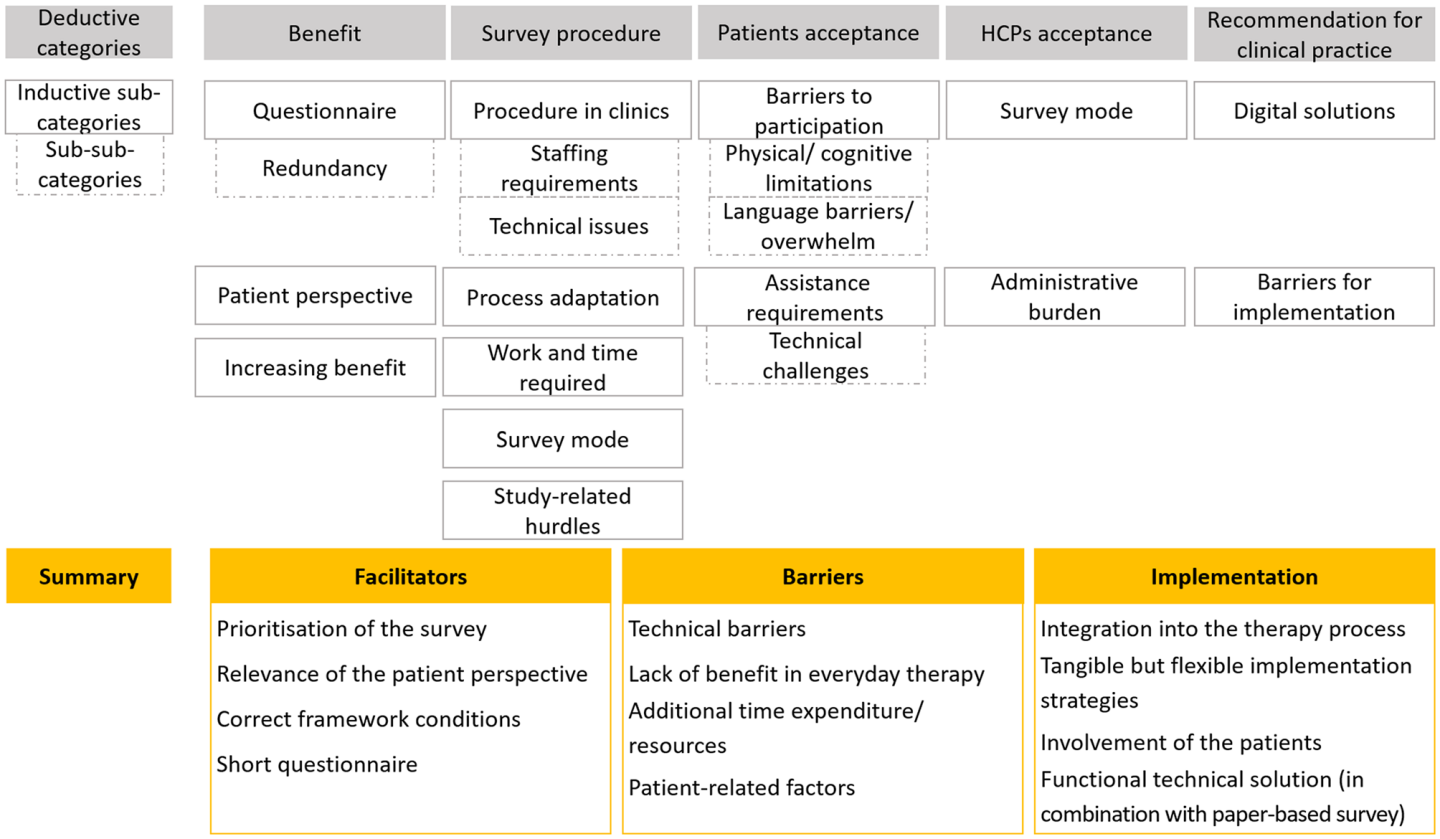
Deductive and inductive categories and summary of the qualitative results

A total of five main categories were formed, each comprising two to five subcategories. For four of these subcategories, up to two sub-subcategories were added to obtain a more differentiated picture of the interview statements (Fig. 5). Finally, the content in the categories was assigned to facilitating factors, barriers, and implementation suggestions of the PROMIS GH-10 in clinical practice (Fig. 5 and Supplementary material 4).

A key facilitator for the successful conduct of the PROMIS GH-10 in clinical practice was providing dedicated time and a calm setting for completion, along with ensuring that patients understood the importance of their participation. The short completion time (around 3–6 minutes) was generally well received. *“The goal was that patients really have time for the questionnaire […] we created a time slot so they could actually fill it in.” (GI1).* Barriers included poor digital integration, tablet usability issues, patient reluctance or inability to use technology (especially in geriatrics), and the additional workload—sometimes up to 30 minutes per patient—without sufficient resources. Some questioned its value for routine care if results were not integrated into treatment. *“There were very few who could fill it out on the tablet […] many were tired, didn’t want to read on the tablet, and the font was small.” (EI2).* Interviewees proposed embedding the process into existing workflows, avoiding redundancy with other questionnaires, ensuring paper-based options, improving technical reliability and integration, and giving patients feedback, potentially in multiple languages. *“If the digital tool were already in the clinical information system […] it would be much easier than doing it separately on an iPad.” (FG1).*

## Discussion

### Key results

The aim of this pilot project was to evaluate the responsiveness and feasibility of PROMIS GH-10 for quality measurements across nine inpatient rehabilitation domains. The PROMIS GH-10 detected statistically significant improvements in HRQOL from admission to discharge in all domains. For the PHS, medium to high effects were found (d = 0.68, by rehabilitation domain 0.50–0.95) whereas the MHS showed small to high effects (d = 0.48, by rehabilitation domain 0.36–1.19).

Assistance requirements varied widely: the highest proportions of patients requiring help to complete the questionnaire were in geriatric and neurological rehabilitation, while psychosomatic rehabilitation had considerably lower proportions.

Qualitative interviews indicated that clinics often adapted data collection processes to local conditions, which sometimes required additional staff resources. Commonly cited facilitators for implementation included the short length of the questionnaire and the perceived value of incorporating the patient perspective.

### Interpretation

The quantitative findings reflect the diversity of the rehabilitation domains and indicate that the PROMIS GH-10 is methodologically suitable as a generic instrument for detecting changes in HRQOL in different rehabilitation contexts.

The observed effect sizes predominantly indicate sufficient responsiveness of the instrument, especially for the PHS [24]. Determining whether these differences are clinically relevant was beyond the scope of this pilot project. However, Terwee et al. [25] cite T-score differences of 2 to 6 for meaningful change for most PROMIS instruments. This is also in line with PROMIS GH-10 specific literature: Khalil et al. [26] report minimally clinically important differences (MCID) for the PHS between 2.3 and 2.5 for patients after knee arthroplasty, depending on the calculation method (distribution vs. anchor-based), while Spiering et al. [27] found corresponding PHS MCIDs between 3.0 and 6.6 and for MHS between 4.3 and 1.6 for baseline and 1-year follow-up in US-based samples for knee arthroplasty. Notably, the anchor-based MCID for the MHS is much smaller than the distribution-based, which means that patients, when asked about meaningful change, tend to perceive smaller changes on the MHS as significant compared to what the distribution-based method suggests. Further studies on specific populations are still needed to go beyond these rules of thumb.

Another way to interpret T-score values in relation to general health (poor, fair, good, very good, excellent) is outlined on the PROMIS website (score cut points). According to their guidelines, the fair-good threshold for PHS is 42 and for MHS it is 40, while the good-very good cut-off is set at 50 for PHS and at 48 for MHS. This would mean that the PHS at admission in this study is below the fair-good cut-off in all rehabilitation domains and above it at discharge (except for the paraplegic rehabilitation), while the MHS is already in the “good” range in all rehabilitation domains at admission (again, except for paraplegic rehabilitation).

The different effect sizes in rehabilitation domains emphasize the relevance of assessing both the physical and mental aspects of HRQOL separately. This is particularly relevant as many outcome measures for rehabilitation have specific objectives regarding physical or mental health [28, 29]. The two-factor structure of the PROMIS GH-10 thus appears to be differentiated enough in order to depict the multidimensional construct of HRQOL.

One finding that requires further investigation is the low effect sizes observed in the musculoskeletal and paraplegic rehabilitation domains on the PHS, while other rehabilitation domains (such as cardiology or pulmonology) show significantly higher effects. There are several possible explanations for this: the presence of more complex or chronic conditions in musculoskeletal and paraplegic patients, making significant improvements harder to achieve; and the limitations of the generic measure, which may not be sensitive enough to detect subtle functionality improvements in these areas. Additionally, differences in rehabilitation approaches, and different patients’ expectations for improvement after acute versus chronic conditions, could also play a role. However, other studies using the VR-12, a 12-item generic PROM of HRQOL, which, like the PROMIS GH-10, provides separate physical and mental health subscores, in German rehabilitation settings show a different pattern, where musculoskeletal rehabilitation demonstrates the largest effect sizes on the physical subscore, while patients with depression score the lowest [30]. The differing effect size patterns compared to German VR-12 studies may be partly due to patient differences: orthopaedic patients in German rehabilitation are on average about 20 years younger than in our Swiss PROMIS sample, which could explain larger physical improvements. Additionally, the VR-12 focuses more on role functioning, potentially making it more sensitive to changes in musculoskeletal rehabilitation than the more general physical health captured by the PROMIS GH-10. A further reason for the larger physical health effect sizes observed in psychosomatic rehabilitation in Switzerland may be the substantial improvements in symptoms such as fatigue and physical distress, which are well captured by the PROMIS GH-10 physical subscale. In general, response shift, where changes in patients’ self-evaluations impact the perceived effects, cannot be ruled out [31].

The interpretation concerning feasibility draws on a mixed-methods approach integrating various data sources, comprising not only the key results but also data quality and field notes from monitoring and support activities. The quantitative findings on assistance requirements indicate diverse domain-specific barriers and for some domain’s considerable additional effort for the clinical staff in administering PROMs. The finding that reading out questions and answers was the most common type of assistance required (about half of all cases across rehabilitation domains and time points) highlights opportunities for technical solutions, such as speech-supporting technologies [32].

However, our data does not reflect that the PROMIS GH-10 led to the numerous missing measurements. HCPs were asked to record the reasons for missing measurements despite patients’ consent. Those reasons were available for only one in five incomplete cases. Both the field notes and the interviews suggest that these were primarily due to clinic-specific processes rather than to the instrument itself or to patients’ refusal to undergo an additional measurement.

Some difficulties in this project arose from structural barriers/operational constraints, such as the lack of digital networking in the hospital system, as revealed in the interviews. In principle, however, there were only a few indications that the PROMIS GH-10 was not practicable as a tool. Occasionally, the desire for an indication-specific tool was expressed to increase clinical usability. This is the major trade-off between a generic instrument that is as short as possible and a longer, disease-specific instrument. The latter can provide greater usability for the treatment and communication process with patients, as already discussed during the PROMs selection process and in the literature [33, 34]. In other words, it is generally only possible to maximise two of the following at the same time: the breadth of health aspects covered (measurement range), the accuracy of measurement (precision), and the brevity of the questionnaire (number of items) [35]. Improving one aspect often requires compromising on another. Further results of the qualitative interviews on barriers and facilitating factors, such as technical administration or clarification of a responsible person, are in line with other international research [12, 36–38]. The intensive use of both survey modes (tablet 60.4%/paper 39.6%) in all rehabilitation domains illustrates how relevant it is to continue to offer paper-based surveys. According to other research, the combination of paper and electronic questionnaires is crucial, for example, to ensure that the implementation does not create bias by disadvantaging populations with lower digital literacy [39–41].

### Generalisability

The results of the study provide insights into rehabilitation in Switzerland as a whole, as it included 28 clinics from three different language regions and all rehabilitation domains. However, potential PROM-specific bias sources, such as (self-)selection or nonresponse, should be considered. The OECD PaRIS study supports the validity of this pilot study data, as it reported similar mean scores for the PHS (45.4) and MHS (47.8) for people living with chronic conditions in primary care in Switzerland [42], aligning with the discharge values in the current study (PHS: 44.4; MHS: 48.7). However, generalisability to other countries is limited due to differences in rehabilitation systems and potential selection effects. For Germany, whose rehabilitation system is the most similar in an international comparison, partly comparable PROMIS GH-10 data for neurological rehabilitation after stroke are available [43]. Apart from that, the authors are not aware of any similarly detailed studies on the PROMIS GH-10 or a similar PROM that report effect sizes across different rehabilitation domains.

Nevertheless, similar challenges in implementing PROMs are also likely to occur in other countries [38]. Generic instruments have the potential to enable both international and cross-sector comparisons, further emphasising their value.

### Strengths and limitations

The strength of the study lies in its successful piloting of the PROMIS GH-10 across nine rehabilitation domains in three language regions at a national level. In addition, the number of cases achieved was sufficient for the planned statistical calculations.

Furthermore, the selection of the questionnaire to be used was decided by the QA using a multi-stage selection process, which is one of the most important steps in the use of PROMs [44].

Another strength is the detailed assessment of assistance requirements, which allows for further aspects relevant to the implementation of PROMs. The database was extended to all available cases with documented assistance requirements, regardless of data completeness (*n* = 2,015 admission and *n* = 1,290 discharge measurements). This approach ensures that the analysis captures the entire spectrum of reported assistance needs, rather than being restricted to complete cases. By leveraging all available information, the analysis minimises potential bias and provides a more comprehensive understanding of the assistance required across the study population.

Despite the positive results, this study is subject to limitations.

First, it cannot be ruled out that other clinics in individual rehabilitation domains with a different patient mix would have resulted in different scores. The pilot clinics voluntarily agreed to participate, which indicates a high level of commitment and potential research interest. Nevertheless, the results of this Study are similar to the OECD Paris Survey suggesting that our sample is comparable to the population in Switzerland [42].

With 866 cases (41.6%), there was a high number of incomplete cases. However, the majority of these incomplete cases in this pilot study resulted from missing discharge measurements (30.1%), while the admission measurement was complete. No clear patterns in the response behaviour were identified, so that no selection bias between rehabilitation domains can be assumed. No rehabilitation domain-specific issues were found that would challenge the suitability of the PROMIS GH-10 for the use in individual rehabilitation domains.

Additionally, in the study population (Supplementary material 3), a selection bias is recognisable, particularly regarding the duration of rehabilitation in paraplegiologic rehabilitation. For administrative reasons, the participating clinics only included patients who were discharged earlier than the usual length of rehabilitation in this domain. This selection limited the sample to patients who undergo a second rehabilitation, while patients in the usually longer-lasting first rehabilitation phase typically achieve greater progress.

Thirdly, the limited patient perspective in the interviews represents a limitation. Although telephone-based interviews were attempted as part of the study, they were discontinued after three sessions because the patients did not recall the questionnaire. This is due to the brevity of the questionnaire and the number of questionnaires to be completed at admission. Therefore, the value of the interviews was limited.

## Conclusion

In summary, the pilot project shows statistically significant pre-post effects of HRQOL in all nine rehabilitation domains, which supports the responsiveness of the instrument for measuring changes in HRQOL in different rehabilitation contexts. Concerning feasibility in clinical practice, the findings point to both facilitating factors and notable challenges, such as the high assistance requirements in some rehabilitation domains.

This pilot project was able to provide important insights into the suitability of the PROMIS GH-10 as an outcome indicator for the Swiss rehabilitation measurement plan.

Emphasising the patient perspective as a national outcome parameter, this pilot project reflects an expansion from primarily functional and performance-based outcome measures to include patient-reported health aspects as a complementary enrichment of quality assessment. By systematically capturing patients’ own perspectives on their health and recovery, this approach supports a more patient-centred evaluation of rehabilitation outcomes. At the same time, it indicates that further methodological refinement and validations are necessary to ensure the meaningful complementary integration of PROMs within quality measurements.

While HRQOL is an indicator that ICF-based rehabilitative treatment should target, the extent of its relevance may vary across different rehabilitation fields.

The results of this pilot project, however, also make it clear that the collection of PROMs is associated with considerable effort for the clinics and should not be an end in itself, which should be taken into account when discussing possible implementation. A sustainable strengthening of the patient perspective through PROMs can only be achieved if the results are incorporated into clinical processes and clinical staff also recognise their added value.

## Electronic supplementary material

Below is the link to the electronic supplementary material.

Supplementary Material 1

Supplementary Material 2

Supplementary Material 3

Supplementary Material 4

## Data Availability

Data is available upon request by submitting an application to the ANQ. Further information can be found on the ANQ-Webportal (https://www.anq.ch/de/downloads/?category=3027%26deepid=b73ef88b1f8e1d540ec8f1d6330c229f).

## Abbreviations

CIRS: Cumulative Illness Rating Scale
d: Effect size
HCP: Health Care Professionals
HRQOL: Health-Related Quality of Life
ICF: International Classification of Functioning, Disability and Health
ICHOM: International Consortium for Health Outcomes Measurement
MCID: Minimally clinically important differences
MHS: Mental Health Summary Score
n: Sample Size
P: p-value
PHS: Physical Health Summary Score
PROM: Patient-Reported Outcome Measure
PROMIS GH-10: Patient-Reported Outcomes Measurement Information System Global Health-10
QA Rehabilitation: Quality Committee Rehabilitation
sd: Standard Deviation
T: T-Score
T1: Admission Measurement
T2: Discharge Measurement
