# Supplementary material for: Strengthening the patient perspective in quality development: a mixed-methods observational study on suitability of the PROMIS GH-10 in Swiss inpatient rehabilitation
Source: BMC Health Serv Res. 2026 Aug 12;26:1141. doi: 10.1186/s12913-026-15291-7 (PMC13479572; doi:10.1186/s12913-026-15291-7)
Supplement: Supplementary file 1 [file 12913_2026_15291_MOESM1_ESM.docx]

*Supplementary material 1: PROMIS GH-10 Items and Scores*

| No. | ID | Item | Relevant for PHS/MHS |
| --- | --- | --- | --- |
| 1 | Global01 | General health | - |
| 2 | Global02 | Quality of life | MHS |
| 3 | Global03 | Physical health | PHS |
| 4 | Global04 | Mental health | MHS |
| 5 | Global05 | Satisfaction Social life | MHS |
| 6 | Global09r | Social role function | - |
| 7 | Global06 | Physical functioning | PHS |
| 8 | Global10r | Emotional problems | MHS |
| 9 | Global08r | Fatigue | PHS |
| 10 | RECODE Global07r | Pain | PHS |
