# Supplementary material for: Strengthening the patient perspective in quality development: a mixed-methods observational study on suitability of the PROMIS GH-10 in Swiss inpatient rehabilitation
Source: BMC Health Serv Res. 2026 Aug 12;26:1141. doi: 10.1186/s12913-026-15291-7 (PMC13479572; doi:10.1186/s12913-026-15291-7)
Supplement: Supplementary file 2 [file 12913_2026_15291_MOESM2_ESM.docx]

Supplementary material 2: Interview guide

Questions

1) **Introduction**

**Short round of introductions**

- **Which patient population do you mainly work with?**
- **What experience do you already have with other research projects in your clinic?**
- **What tasks have you taken on as part of the PROMIS GH-10 pilot project?**

2) **Challenges & potentials**

What was the specific survey situation like at your location? (Where was the survey conducted? What (technical) means were used? At what point did the survey take place (e.g. at the beginning of an interview, at the end)?

Can you describe a typical interview with a patient?

3) **Effort – benefit**

How labour-intensive was the survey per patient?

What did the questionnaire mean in everyday life and what were the benefits?

4) **Practicability & acceptance**

Which patients had particular difficulties with the PROMIS survey?

How was the acceptance of patients and clinic staff?

Did the changes indicated in the questionnaire during rehabilitation correspond to the assessment of the staff and patients?

5) **Concept of PROMs**

Is it important to measure quality of life from the patient's perspective during inpatient rehabilitation?

In your opinion, can the questionnaire contribute to including the patient's perspective more in the treatment?

6) **Implementation**

What ideas do you have for reducing the hurdles for patients when collecting PROMs/the PROMIS GH-10?

What recommendations do you have for implementation in the regular rehabilitation measurement plan?
