# Supplementary material for: Strengthening the patient perspective in quality development: a mixed-methods observational study on suitability of the PROMIS GH-10 in Swiss inpatient rehabilitation
Source: BMC Health Serv Res. 2026 Aug 12;26:1141. doi: 10.1186/s12913-026-15291-7 (PMC13479572; doi:10.1186/s12913-026-15291-7)
Supplement: Supplementary file 3 [file 12913_2026_15291_MOESM3_ESM.docx]

Supplementary material 3: Sample description comparing the national measurement plan sample with two linked pilot subsamples (total and complete cases)*

|  | **Geriatric** | | | | | **Internal medicine** | | | | | | | **Cardiac** | | | | |
| --- | --- | --- | --- | --- | --- | --- | --- | --- | --- | --- | --- | --- | --- | --- | --- | --- | --- |
| **Number of transmitted cases/  linked cases** | **CH-total** | **P-total** | **P-complete** | | | | **CH-total** | | **P-total** | | **P-complete** | | | | **CH-total** | **P-total** | **P-complete** |
|  | 19'790 | 240 | 115 | | | | 4'970 | | 96 | | 46 | | | | 6'483 | 213 | 161 |
| **Gender** (in %) |  |  |  | | | |  | |  | |  | | | |  |  |  |
| male | 36.1 | 41.3 | 45.2 | | | | 49.1 | | 59.4 | | 56.5 | | | | 70.2 | 70.0 | 69.6 |
| female | 63.9 | 58.8 | 54.8 | | | | 50.9 | | 40.6 | | 43.5 | | | | 29.8 | 30.0 | 30.4 |
| **Age (mean, SD in years)** | 83.9 (6.8) | 82.2 (6.0) | 81.9 (5.6) | | | | 68.6 (13.4) | | 68.7 (12.9) | | 66.2 (14.5) | | | | 68.1 (11.5) | 68.5 (11.7) | 66.9 (11.2) |
| **Duration of Rehabilitation (mean, SD in days)** | 22.5 (11.1) | 24.2 (11.1) | 26.2 (11.8) | | | | 22.8 (10.9) | | 20.2 (9.4) | | 23.2 (9.4) | | | | 21.0 (5.9) | 22.2 (8.0) | 22.3 (7.2) |
| **CIRS total score (mean, SD in points)** | 18.2 (6.5) | 21.6 (6.1) | 21.4 (6.4) | | | | 17.6 (7.1) | | 18.7 (6.9) | | 16.9 (6.0) | | | | 17.9 (6.1) | 18.6 (6.7) | 18.7 (6.6) |
|  | **Musculoskeletal** | | | **Neurological** | | | | | | | | | | **Oncological** | | | |
| **Number of transmitted cases/  linked cases** | **CH-total** | **P-total** | **P-complete** | | | | **CH-total** | | **P-total** | | **P-complete** | | | | **CH-total** | **P-total** | **P-complete** |
|  | 27'684 | 497 | 286 | | | | 13'492 | | 182 | | 113 | | | | 1'503 | 75 | 57 |
| **Gender** (in %) |  |  |  | | | |  | |  | |  | | | |  |  |  |
| male | 36.5 | 40.2 | 45.1 | | | | 53.9 | | 51.6 | | 45.1 | | | | 47.8 | 34.7 | 35.1 |
| female | 63.5 | 59.8 | 54.9 | | | | 46.1 | | 48.4 | | 54.9 | | | | 52.2 | 65.3 | 64.9 |
| **Age (mean, SD in years)** | 71.3 (13.2) | 68.0 (13.9) | 65.8 (14.5) | | | | 66.7 (15.2) | | 67.6 (13.6) | | 67.8 (13.4) | | | | 67.8 (12.2) | 65.1 (14.3) | 64.5 (14.2) |
| **Duration of Rehabilitation (mean, SD in days)** | 22.0 (12.8) | 24.8 (12.5) | 25.1 (12.2) | | | | 36.8 (27.6) | | 32.1 (19.0) | | 29.6 (16.5) | | | | 21.0 (8.5) | 20.4 (6.8) | 20.2 (6.5) |
| **CIRS total score (mean, SD in points)** | 13.0 (6.3) | 13.2 (7.1) | 12.5 (7.6) | | | | 14.7 (7.4) | | 14.6 (7.0) | | 13.8 (5.7) | | | | 15.9 (6.4) | 15.7 (8.1) | 14.5 (7.8) |
|  | **Paraplegic** | | | | **Psychosomatic** | | | | | | | **Pulmonological** | | | | | |
| **Number of transmitted cases/  linked cases** | **CH-total** | **P-total** | **P-complete** | | | | | **CH-total** | | **P-total** | **P-complete** | | | | **CH-total** | **P-total** | **P-complete** |
|  | 799 | 70 | 37 | | | | | 2'214 | | 47 | 38 | | | | 4'021 | 149 | 91 |
| **Gender** (in %) |  |  |  | | | | |  | |  |  | | | |  |  |  |
| male | 63.3 | 70.0 | 64.9 | | | | | 34.8 | | 17.0 | 15.8 | | | | 51.6 | 47.0 | 49.5 |
| female | 36.7 | 30.0 | 35.1 | | | | | 65.2 | | 83.0 | 84.2 | | | | 48.4 | 53.0 | 50.5 |
| **Age (mean, SD in years)** | 56.0 (17.0) | 56.0 (14.4) | 53.5 (15.3) | | | | | 51.8 (13.8) | | 52.5 (12.4) | 51.6 (13.2) | | | | 69.4 (10.8) | 68.5 (12.4) | 68.6 (12.1) |
| **Duration of Rehabilitation (mean, SD in days)** | 74.5 (68.2) | 37.7 (16.9) | 36.8 (16.2) | | | | | 24.9 (10.7) | | 28.3 (15.4) | 29.8 (15.9) | | | | 20.4 (7.1) | 23.6 (9.5) | 23.6 (8.4) |
| **CIRS total score (mean, SD in points)** | 19.9 (7.9) | 18.0 (6.5) | 16.2 (6.8) | | | | | 8.3 (4.7) | | 7.5 (4.8) | 6.5 (3.6) | | | | 16.4 (5.7) | 17.2 (5.8) | 16.9 (5.7) |

*CIRS (Cumulative Illness Rating Scale)*

**The two pilot subsamples included here (total and complete cases) represent only those cases from the original pilot dataset that could be linked to the Swiss national measurement plan sample (CH-total). Due to the strict forward-only pseudonymization algorithm used during data processing, only exact matches of patient IDs could be linked. Minor input errors (e.g., digit transpositions) could not be resolved post hoc, resulting in a reduced number of matched cases (n=1’519, 75.4% successful linkage in pilot-total sample; n=944, 77.6% in pilot-complete cases).*
