# Supplementary material for: Strengthening the patient perspective in quality development: a mixed-methods observational study on suitability of the PROMIS GH-10 in Swiss inpatient rehabilitation
Source: BMC Health Serv Res. 2026 Aug 12;26:1141. doi: 10.1186/s12913-026-15291-7 (PMC13479572; doi:10.1186/s12913-026-15291-7)
Supplement: Supplementary file 4 [file 12913_2026_15291_MOESM4_ESM.docx]

Supplementary material 4: Qualitative results with Examples

|  | Original Example | Translation |
| --- | --- | --- |
| Facilitators |  |  |
| Prioritisation of the survey | „Also im zweiten Teil, wo wir den Prozess angepasst haben, haben wir versucht dem Fragebogen eine gewisse Wichtigkeit zu geben und haben gesagt, der Sinn muss erklärt werden. [...] Und dort wurde das dann auch erklärt, warum machen wir den Fragebogen, warum ist ihre Antwort auch so wichtig, warum machen wir das am Anfang und auch nochmal am Ende.“ (EI1) | “So in the second part, where we adapted the process, we tried to give the questionnaire a certain importance and said that the meaning had to be explained. [...] And then we also explained why we were doing the questionnaire, why your answer was so important, why we were doing it at the beginning and again at the end.” |
| Relevance of the patient perspective | „Man muss die Patientenperspektive einbeziehen. Wir integrieren sie bereits sehr stark in die Behandlung, es ist nicht sicher, ob dies daher einen diesbezüglichen Mehrwert darstellt.“ (GI3) | „The patient's perspective must be taken into account. We already integrate it very strongly into treatment, so it is not certain whether this represents added value in this regard.“ |
| Correct framework conditions | „Das Ziel war, dass die Patienten wirklich Zeit haben für den Fragebogen. Weil in der Rehabilitation ist eher alles sehr eng getaktet. Also sie haben sehr viele Termine am Tag und da war die Idee, dass wir uns einen Zeitraum schaffen, damit sie dann wirklich auch den Fragebogen ausfüllen können.“ (GI1) | “The aim was for the patients to really have time to complete the questionnaire. Because in rehabilitation, everything tends to be very tightly scheduled. So they have a lot of appointments every day and the idea was that we would create a period of time so that they could actually complete the questionnaire.” |
| Short questionnaire | „Ja, ich kann mich dem anschliessen, die Befragung selber ging eigentlich nicht so lang. 3-6 Minuten ist glaub ich recht gut.“ (FG1) | “Yes, I agree, the survey itself didn't actually take that long. I think 3-6 minutes is quite good.” |
| Barriers |  |  |
| Technical barriers | „Es gab sehr wenige, die den Fragebogen mit dem Tablet ausfüllen konnten. Bei der Geriatrie wiederum gar niemand. Dort musste ich es ausfüllen, entweder haben die Hände gezittert oder sie wussten nicht, wie es geht oder waren allgemein abgelehnt gegenüber der Technologie und auch die anderen: Viele waren müde, und wollten es nicht auf dem Tablet lesen, die Schrift ist klein und man kann diese auch nicht einstellen.“ (EI2) | “There were very few people who were able to complete the questionnaire on a tablet. In geriatrics, no one at all. I had to fill it out there, either their hands were shaking or they didn't know how to do it or were generally dismissive of the technology and the others: Many were tired and didn't want to read it on the tablet, the font is small and you can't adjust it either.” |
| Lack of benefit in everyday therapy | „Es hat für mich keinen Zusatznutzen. Ich habe das nicht in einem Gespräch vertieft „oh, sie haben ja starke Schmerzen angegeben…“ - das habe ich nicht als Teil meiner Aufgabe gesehen, […] sondern eher im Rahmen des Klinikalltages, mal mit der Stationsärztin angesprochen […], aber ich habe es sonst in meinem Klinikalltag nicht weiterverwendet, daher habe ich es nicht als weiteren Nutzen gesehen für den Klinikalltag.“ (FG1) | “It has no added value for me. I didn't go into it in depth in a conversation ‘oh, you've reported severe pain...’ - I didn't see it as part of my job, [...] but rather in the context of everyday hospital life, sometimes discussed with the ward doctor [...], but otherwise I didn't use it in my everyday hospital life, so I didn't see it as an additional benefit for everyday hospital life.” |
| Additional time expenditure/ resources | „zwischen den Ärzten, die Informationen weitergeben haben, der Forschung, die uns geholfen hat, dem Pflegepersonal, das die Fragebögen ausgehändigt hat und die Antworten in Excel und in das iPad eingegeben hat, war der Aufwand ziemlich enorm.“ (E4) | „Between the doctors who shared information, the researchers who helped us, and the nursing staff who handed out the questionnaires and entered the answers into Excel and the iPad, the effort involved was quite enormous.“ |
| Patient-related factors | „Bei den Geriatriepatienten hat es eine ganze Weile länger gedauert, d.h. es hat auch schon Mal eine halbe Stunde gedauert, und sobald die Patient:innen etwas angeschlagen waren, oder nicht gut aufgelegt oder die Einverständniserklärung genau durchlesen wollten, dann haben die fünfzehn Minuten nicht gereicht.“ (EI2) | “With geriatric patients, it took quite a bit longer, sometimes up to half an hour, and as soon as the patients were feeling a bit under the weather, or were not in a good mood, or wanted to read the consent form carefully, then fifteen minutes was not enough.” |
| Implementation |  |  |
| Integration into the therapy process | „Ich denke die Lebensqualität ist wichtig zu erfassen […], aber nicht dreimal hintereinander, man kann das effizienter gestalten. […]. Das würde auch dazu führen, dass der Patient nicht beelendet wird und auch das Personal nicht. Weil es könnte sich dann auf anderes Wesentliches konzentrieren. Im Moment sind wir eine „Abfrögli-Nation“ zurzeit. In ganz vielen Facetten und wir fragen uns zu Tode, aber wir gehen nicht auf die Effekte nachher ein.“ (GI2) | “I think the quality of life is important to capture [...], but not three times in a row, you can do it more efficiently. [...]. This would also mean that the patient would not be distressed and neither would the staff. Because they could then concentrate on other essentials. At the moment, we are an ‘Abfrögli nation’. In many facets and we're asking ourselves to death, but we're not looking at the effects afterwards.” |
| Concrete but flexible implementation strategies | „Es ist immer wichtig, die Lebensqualität zu bewerten, aber nicht in dieser Form. Es würde mehr Sinn machen, dies beim Austritt und einige Wochen danach zu tun.“ (GI3) | “It is always important to assess quality of life, but not in this way. It would make more sense to do this at discharge and a few weeks afterwards.” |
| Involvement of the patients | „[…] eine Auswertung [wäre] toll oder ein gewisses Feedback […] oder vielleicht kann man auch den Patienten so ein Feedback per E-Mail schicken.“ (EI1) | “[...] an evaluation [would be] great or some feedback [...] or perhaps you can also send the patients such feedback by e-mail.” |
| Functioning technical solution (in combination with paper-based survey) | „Wenn das Digitale schon im normalen KIS oder so erfasst wäre, dass man bestimmte Daten direkt da rausziehen könnte, dann wäre es schon viel viel einfacher, als das noch zu machen auf dem iPad.“ (FG1) | “If the digital data was already recorded in the normal HIS or in such a way that you could pull certain data directly from there, then it would be much easier than doing it on the iPad.” |
